# Assessing and Improving the Knowledge of Sexually Transmitted Infections among High School Adolescents

**DOI:** 10.1155/2021/6696316

**Published:** 2021-04-09

**Authors:** Itisha S. Jefferson, S. Kayo Robinson, Eleanor Tung-Hahn, Roan Schumann, Synthia Marrero-Conti, Jasmine M. Walton, Eileen Golden, Emily Poon, Murad Alam, Rebecca Tung

**Affiliations:** ^1^Stritch School of Medicine, Loyola University, Chicago, IL, USA; ^2^College of Public Health, The University of South Florida, Tampa, FL, USA; ^3^Robins School of Business, University of Richmond, Richmond, VA, USA; ^4^East Ridge High School, Clermont, FL, USA; ^5^Oak Street Health, Chicago, IL, USA; ^6^Department of Obstetrics and Gynecology, Cleveland Clinic, Cleveland, OH, USA; ^7^Department of Dermatology, Feinberg School of Medicine, Northwestern University, Chicago, IL, USA; ^8^Florida Dermatology and Skin Cancer Centers, Winter Haven, FL, USA

## Abstract

**Background:**

According to the Center for Disease Control and Prevention (CDC), half of the 20 million new sexually transmitted infections (STIs) occur annually in youth aged 15 to 24. It is critical for dermatologists to be able to provide young patients with accessible education about how to identify, prevent, and treat these conditions. Our pilot study sought to assess the impact of a live presentation about STIs in high school students and to evaluate students' baseline and acquired knowledge about common STIs using a before and after survey.

**Methods:**

This survey study was conducted as part of the health education curriculum at a suburban high school. An interactive scenario-based presentation about STIs was given to participants. Identical, deidentified preintervention and postintervention surveys were completed by subjects to assess their baseline and acquired knowledge of STIs. Each question was worth 1 point, for a total of 8 points.

**Results:**

74 high school students were surveyed. Overall, there was a mean improvement of 1.85 points in the posttest score in comparison to pretest score with a standard deviation of 1.58 (*p* < 0.0001). Among all participants, the mean pretest score was 1.07 (SD = 0.75) and the mean posttest score was 2.92 (SD = 1.59).

**Conclusions:**

This study demonstrated that many young adults are unaware of the common types of STIs, symptoms, and potential complications. While our scenario-based presentation was effective at providing understandable content to help improve students' knowledge regarding STIs, additional educational resources in varied formats could likely further these gains.

## 1. Introduction

The Center for Disease Control and Prevention (CDC) estimates that half of the 20 million new sexually transmitted infections (STIs) occur annually in youth aged 15–24, who make up just over one quarter of the sexually active population in the United States [1]. Prevalence of STIs is higher in sexually experienced young females compared to males [2]. This marked difference is thought to be due to anatomic differences and relatively fewer bothersome symptoms in females who are infected with common STIs such as chlamydia and gonorrhea [3]. Although STIs can affect people of all ages, they can have more dire consequences and long reaching effects in young adults.

As a group, young people are more likely to participate in high-risk behaviors, sex without protection and having multiple sex partners [4]. These risky behaviors are more evident in this cohort due to evolving brain development and executive decision making [4, 5]. Additionally, teens are less likely to seek medical attention and utilize sexual health services than adults due to multiple barriers: lack of accessibility to services, concerns about confidentiality, stigma attached to seeking STI services, and limited knowledge about STIs, their signs and symptoms and delayed treatment complications [6, 7].

Although STIs are commonly perceived as purely gynecologic or urologic conditions, many STIs have dermatologic manifestations [8]. Since many STIs can result in severe medical consequences such as infertility, chronic pain, adverse pregnancy outcomes, cancer, and, in some cases, death [4], it is critical for the dermatologist to be able to have an open dialogue with young patients about STIs as well as being able to provide meaningful and accessible education about how to identify, treat, and prevent these same conditions.

The aims of our pilot study are to assess the impact of an interactive scenario-based presentation about STIs and dermatologic skin manifestations in high school students and to evaluate students' baseline and acquired knowledge about common sexually transmitted infections using a before and after survey.

## 2. Methods and Statistical Analyses

This study was conducted as a part of the health education curriculum in collaboration with educators at a suburban high school. All parents were notified in advance and were given the opportunity to opt in or to opt out their child's attendance and participation in this sexually transmitted infections (STIs) lecture and study.

An identical, deidentified pre- and postintervention survey (10 questions with multiple choice answers—2 questions focused on demographics and 8 questions pertained directly to the lecture content) was given to participants to assess both their baseline and acquired knowledge of STIs (Supplement 1). Questions relating to types of common STIs, signs and symptoms of STIs, and complications of STIs allowed for choice of more than one answer. During their scheduled class time, students first completed the before survey (10 minutes), viewed the interactive 25-minute PowerPoint live presentation lesson, and then took the after survey. Each content question was worth 1 point, for a total of 8 points for the before survey as well as the after survey. This curriculum was taught to three classes of students, comprised of 11th and 12^th^ graders. Content for this lesson was drawn from multiple sources and highlighted the most common STIs, major signs and symptoms of STIs, treatment of STIs, and complications if left untreated. To introduce each STI condition, students were informally queried as a group as to whether they were aware of the typical disease presentation and any questions were answered. After the presentation, an identical survey questionnaire was administered. We chose to include one question which elicited where students had previously received information on STIs.

Data was analyzed by SAS Studio 3.8 (Enterprise Edition). All statistics were generated using this program. Analysis consisted of paired sample *t*-test comparing numerically rated answers on the before test and after test. For all two-tailed tests, significance was set at 0.05.

## 3. Results

74 students (57 eleventh graders and 16 twelfth graders and 1 unspecified) participated in this educational intervention study (Table 1). Mean age was 16.48 [standard deviation (SD) of 0.58]. Students reported their knowledge on STIs prior to the educational session (Table 2). Among participants, 95% had heard of STIs before, while 4% had never heard about STIs, and 1% indicated they do not know. With regard to sources where students stated they had received information about STIs, the majority identified school (91%) followed by the Internet (72%) and family (60%). Few students cited periodicals including magazines (4%) and television/radio (5%) as sources of STI information. For this question, students could select multiple sources, if applicable.

Table 3 demonstrates that survey scores of all students significantly improved after viewing this presentation. The maximum score attainable on the pre- and posttest evaluation was 8. Participants were given a score of 1 for answering a single question correctly or a score of 0 for answering a single question incorrectly. No partial credit was given. Overall, there was a mean improvement of 1.85 points in their posttest score as compared to their pretest score with a standard deviation of 1.58 (*p* < 0.0001). For all participants regardless of grade, the mean pretest score was 1.07 (SD = 0.75) and the mean posttest score was 2.92 (SD = 1.59).

Subgroup analysis revealed the mean overall pre- and posttest scores for Grade 11 participants were 1.09 (SD = 0.79) and 3.11 (SD = 1.62), respectively, with a mean improvement of 2.02 (SD = 1.65) (*p* < 0.0001). Average percentage improvement in scores was 185% for Grade 11 students. For students in Grade 12, the mean overall pre- and posttest scores were 1.00 (SD = 0.63) and 2.25 (SD = 1.34) with a mean improvement of 1.25 (SD = 1.18) (*p*=0.0007). The average percentage improvement in scores was 125% for Grade 12 participants.

Finally, with respect to the before versus after answers for each individual question, all students significantly improved in their ability to answer correctly following participation in the STI session. However, with regard to questions 3 (what are the main signs and symptoms of sexually transmitted infections/diseases?) and 8 (which of the following conditions can be treated with cryosurgery (freezing)?), score improvement was noted, yet it was not significant (Table 4). Students achieved a mean pretest score of 0.44 (SD = 0.5) and posttest score of 0.58 (SD = 0.5) (*p*=0.06) on question 3. Most students answered question 8 (regarding Human Papillomavirus treatment) incorrectly. On this question, students achieved a mean pretest score of 0.05 (SD = 0.23) and posttest score of 0.14 (SD = 0.34) (*p*=0.08).

## 4. Limitations

The primary limitation of this study is that it was a small pilot study conducted at a single site. The results found herein may not be representative for other institutions or geographic locations. Educational institutions have different requirements regarding presentation and format of sexual education topics.

Although statistically significant improvement in the overall score amongst all students in both grades was noted, the mean increase (1.85) was still relatively low following participation in the session. This suggests that there is room for further refinement and modification of the content and delivery of the information presented. Possibilities include emphasizing take-home points in a summary slide following each disease topic, incorporating a final review slide at the conclusion of the session, either condensing or dividing the education session into two or more separate sessions given the density of the content material, and making the presentation available electronically for later retrieval and reference. Furthermore, gathering feedback from the students following the session could also provide insight on ways future educational sessions could be improved. As described earlier, students achieved only modest improvement in their scores for questions 3 and 8. Further simplification and repetition of the specific content may be helpful for learners. Additionally, the question format could also be altered to better capture whether or not knowledge about these conditions was acquired. As a whole, the information covered in the STI presentation may have been new for our participants. Allowing students to have electronic access to the presentation after the session for review could also be beneficial.

We recognize that Grade 11 students were more highly represented compared to Grade 12 students. Certainly, equal representation would further strengthen our study. We have reported the immediate effects of knowledge improvement following our session by utilizing the before test, but do not have data on the long-term impact secondary to pandemic-related school closures. Finally, it may be advantageous to assess whether or not gender differences affect study results. Comparing various delivery methods of STI education (live presentation, online video format, and written material) may also prove useful for optimizing teaching about this subject. It is also important to keep in mind that administration and teacher collaboration as well as parental agreement are always needed in conducting any type of study with minors on this topic.

## 5. Discussion

Half of the annual STI cases in the United States occur in adolescents and young adults [1]. Because this population is disproportionately affected by STIs, we created an educational workshop in hopes of educating students about these potentially life altering conditions. In 2016, the CDC reported that 81.6% of high school districts had adopted a policy to teach specific health topics including STI prevention [9]. Districts were required to teach about substance use prevention, mental health, nutrition, and violence prevention in addition to STI education. This policy was enforced by requiring assessments measuring student achievement based on the health education program. However, only 51.6% of these districts actually implemented a school-based health program for their high school students [9]. It was reported that increased staffing and professional development of health teachers could further improve the current state of high school health education and ensure students are being taught these topics. In addition, the CDC recommended adjunctive resources such as The National Health Education Standards, the National Sexuality Education Standards, and the Division of Adolescent and School Health (DASH) that could be utilized by health educators for teaching STI prevention, knowledge, and skills [10].

In an interview format study, Almeida et al. found that preventative sexual health education implemented in the school system is beneficial for young adults [11]. They interviewed 22 high school students about their knowledge of STIs, AIDS, pregnancy, and the role of school in sex education. They found that students reported their teachers to be their first choice as a source of STI information [11]. Students also recognized the role of their school lectures and family cooperation in health promotion. Similarly, in our study, participants stated that their school served as the primary source of information on STIs. Taken together, these findings underscore the important role that schools have in providing much needed education about STIs.

In a structured questionnaire format study, Nguyen et al. investigated knowledge about STIs among adult Vietnamese dermatology patients. They found that participants had limited knowledge about STIs including common presenting symptoms and available prevention strategies [12]. Of interest, their study revealed that younger participant age and residential status of living with a spouse or a partner were associated with increased knowledge about STIs. Further, they found that the Internet, social media, and healthcare providers were primary sources of STI information [12]. This speaks to the important role that healthcare providers such as dermatologists can play as educators to raise awareness about STIs in patients and community members. Harnessing technology such as the Internet and social media platforms can also extend the reach of a health care team to provide accessible, accurate, and always available content about STIs to all.

Jones and colleagues suggested that social media and text messaging may be promising approaches for effectively increasing STI knowledge among both young men and women [13]. Out of the 11 studies that were evaluated, seven studies examined the effectiveness of social media or texting interventions on STI knowledge. All of them showed significant increases in STI knowledge, including increased understanding of sexual protection methods and transmission modes, following an instructional intervention [13]. Although social media is an easy and effective way to circulate information, there are some potential concerns to consider if it is used for a teaching platform about this topic. Some potential issues include but are not limited to informed consent to participate by either the subject or parents if the subject is a minor, participant privacy and confidentiality, protection of site content and prevention of inappropriate postings, and secure storage of participant contact information for future followup [13, 14].

Information dissemination is one thing but making content “stick” in the minds of learners is quite another. Malik and colleagues sought to determine whether a lecture accompanied by pretest/posttest teaching model versus a post-test-only model was more advantageous in terms of learning outcomes. They found that a pretest/posttest model was more effective than a lecture followed by a posttest alone [15]. They concluded that students, when given the opportunity to identify difficult topics beforehand with the pretest, were better able to adjust their attention to facilitate comprehension during the lecture, which ultimately resulted in improved knowledge acquisition outcomes [15]. With this in mind, our study utilized the pretest/posttest teaching model for the dual purposes of achieving effective learning outcomes and assessing students' baseline knowledge about STIs.

Another effective teaching method, termed spaced repetition, has been shown to improve learning outcomes and long-term retention [16, 17]. Spaced repetition is a memorization technique in which educational content is reviewed repeatedly on a particular schedule, which is based on a spaced repetition algorithm [17]. Kang highlighted using spaced repetition in the classroom setting. They found that when content was repeated over months as part of teaching curriculums, students were better learners. Additionally, they concluded that computerized instruction could provide an ideal means for delivery of spaced repetition within the classroom [16]. In the case of STI education, combining in-person interactive STI teaching models with follow-up e-learning, which incorporates virtual presentation of the material multiple repetitions over a period of time, could optimize students' learning outcomes. The challenge, of course, is having allocated time to permit this type of multimodal instruction.

Because STIs commonly present with cutaneous manifestations and can have serious health ramifications if left unidentified and untreated, we in dermatology can extend our reach beyond the clinical setting by collaborating with educators to chart a new course for young adults by providing effective accessible education which promotes STI awareness, prevention, detection, and treatment. McCaw highlighted the longstanding connection between dermatology and venereology dating back to the sixteenth century. At that time, it was Syphilis, often first recognized by its cutaneous findings, that propelled the expanding frontier of dermatology. Two centuries later, another STI, Herpes, was recognized in the dermatologic realm as a communicable disease with cutaneous manifestations [18].

Historically, the specialty of dermatology was previously known as dermatology and syphilology [19, 20], but syphilology was dropped due to effective treatment regimens and the evolving broader scope of dermatologic practice [19]. The profound impact of dermatology on STI disease characterization and treatment should not be lost. Rather, it is important for modern dermatologists not only to be comfortable with the diagnosis and treatment of STIs but also to advocate for and advance STI education among young adults with the goals of prevention and early detection.

## 6. Conclusion

This pilot study demonstrated that many young adults are unaware of the common types of STIs, symptoms, and potential complications. Although students' knowledge of STIs did increase after participating in our live interactive scenario-based presentation, more can be done on this educational front. Design of future educational tools could also incorporate other teaching modalities such as e-learning in some form, which can promote longer-term retention of STI content and may ultimately influence behaviors that impact health of both the individual and the public.

## Figures and Tables

**Table 1:** Demographics (grade and age).

*Grade*		Count	%
	11	57	77.02703
	12	16	21.62162
	N/A	1	1.351351

*Age*		Mean	SD
		16.48	0.58

**Table 2:** Prior STD and STI knowledge.

	Answer	Count	%
*Have you ever heard of Sexually Transmitted Diseases (STDs) or Sexually Transmitted Infections (STIs)?*	Yes	70	94.59459
	No	3	4.054054
	Don't know	1	1.351351

*From where have you received information on STDs or STIs?*			
	School	67	90.54054
	Internet	53	71.62162
	Family	44	59.45946
	Friends	38	51.35135
	Hospital/Clinic	31	41.89189
	Television	23	31.08108
	Youth club	6	8.108108
	Radio	4	5.405405
	Magazine	3	4.054054
	N/A	2	2.702703

**Table 3:** Participant group scores before and after STI presentation.

Participant groups	n	Pretest score		Posttest score		Mean diff	SD of mean diff	*p* value
		Mean	SD	Mean	SD			
Grade 11	57	1.09	0.79	3.11	1.62	2.02	1.65	<0.0001
Grade 12	16	1	0.63	2.25	1.34	1.25	1.18	0.0007
All participants	74	1.07	0.75	2.92	1.59	1.85	1.58	<0.0001

**Table 4:** Scores of each individual question before and after STI presentation.

Question	Pretest score		Posttest score		Mean diff	SD of mean diff	*p* value
	Mean	SD	Mean	SD			
1	0	0	0.23	0.42	0.23	0.42	<0.0001
2	0	0	0.16	0.34	0.14	0.34	0.0012
3	0.44	0.5	0.58	0.5	0.14	0.6	0.06
4	0.08	0.27	0.62	0.49	0.54	0.5	<0.0001
5	0.35	0.48	0.66	0.48	0.28	0.59	<0.0001
6	0.01	0.12	0.19	0.39	0.18	0.42	0.0005
7	0.09	0.29	0.38	0.49	0.28	0.51	<0.0001
8	0.05	0.23	0.14	0.34	0.08	0.4	0.08

## Data Availability

Data are available on request. Requests for access to data should be made to Dr. Rebecca Tung, MD, at drrebeccatung@gmail.com.

## Supplementary Materials

Supplementary Materials An identical, deidentified preintervention and postintervention survey (10 questions with multiple choice answers—2 questions focused on demographics and 8 questions pertained directly to the lecture content) was given to participants to assess both their baseline and acquired knowledge of STIs (Supplement 1).
