# Supplementary material for: Assessing and Improving the Knowledge of Sexually Transmitted Infections among High School Adolescents
Source: Dermatol Res Pract. 2021 Apr 9;2021:6696316. doi: 10.1155/2021/6696316 (PMC8057898; doi:10.1155/2021/6696316)
Supplement: Supplementary Materials — An identical, deidentified preintervention and postintervention survey (10 questions with multiple choice answers—2 questions focused on demographics and 8 questions pertained directly to the lecture content) was given to participants to assess both their baseline and acquired knowledge of STIs (Supplement 1). [file 6696316.f1.docx]

Supplement 1

Question and Answer Legend:

1. Please choose which diseases are Sexually Transmitted Infections/Diseases (STIs/STDs)? **Syphilis, Chlamydia, Gonorrhea, HIV/AIDS, Herpes, Trichomoniasis, Human Papillomavirus (HPV)**
2. What are the 3 most common Sexually Transmitted Infections/Diseases in adolescents? **Chlamydia, Gonorrhea, Human Papillomavirus (HPV)**
3. What are the main signs and symptoms of Sexually Transmitted Infections/Diseases? **Discharge from penis, Rash, Burning pain on urination, Genital ulcers or open sores**
4. Which of the following can frequently disguise itself as other infections/diseases on the skin (also frequently known as the ‘great masquerader’)? **Syphilis**
5. Which STI/STD has a vaccine available to prevent it? **Human Papillomavirus (HPV)**
6. What are the complications of STIs/STDs if left untreated? **Cancer, Infertility, Ectopic Pregnancy, Death**
7. Which of the following is a chronic (longstanding) cutaneous (appears on the skin) viral sexually transmitted disease/infection that can be suppressed with medications? **Herpes**
8. Which of the following can be treated with cryosurgery (‘freezing’)? **Human Papillomavirus (HPV)**

**Supplementary Description**

An identical, de-identified pre- and post-intervention survey (10 questions with multiple choice answers-- 2 questions focused on demographics and 8 questions pertained directly to the lecture content) was given to participants to assess both their baseline and acquired knowledge of STIs (Supplement 1).
